# Enhancement in Mechanical and Shape Memory Properties for Liquid Crystalline Polyurethane Strengthened by Graphene Oxide

**DOI:** 10.3390/polym8070236

**Published:** 2016-07-19

**Authors:** Yueting Li, Huiqin Lian, Yanou Hu, Wei Chang, Xiuguo Cui, Yang Liu

**Affiliations:** 1 Beijing Key Lab of Special Elastomer Composite Materials, College of Materials Science and Engineering, Beijing Institute of Petrochemical Technology, Beijing 102617, China; liyueting@bipt.edu.cn (Y.Li); huyanou@bipt.edu.cn (Y.H.); changwei@bipt.edu.cn (W.C.); liuyang@bipt.edu.cn (Y.Liu); 2 College of Materials Science and Engineering, Beijing University of Chemical Technology, Beijing 100029, China

**Keywords:** graphene oxide, liquid crystalline polyurethane, composites, shape memory property

## Abstract

Conventional shape memory polymers suffer the drawbacks of low thermal stability, low strength, and low shape recovery speed. In this study, main-chain liquid crystalline polyurethane (LCPU) that contains polar groups was synthesized. Graphene oxide (GO)/LCPU composite was fabricated using the solution casting method. The tensile strength of GO/LCPU was 1.78 times that of neat LCPU. In addition, shape recovery speed was extensively improved. The average recovery rate of sample with 20 wt % GO loading was 9.2°/s, much faster than that of LCPU of 2.6°/s. The enhancement in mechanical property and shape memory behavior could be attributed to the structure of LCPU and GO, which enhanced the interfacial interactions between GO and LCPU.

## 1. Introduction

Shape-memory polymers (SMPs) are smart materials that have attracted much attention in fields ranging from the scientific to the industrial. SMPs are good candidates for clothing manufacturing, deployable space structures, morphing aircraft, and medical treatment due to their excellent structural versatility, high shape recovery ratio, easy processing, and low cost [1]. SMPs have the ability to memorize a permanent shape and to be programmed for one or many temporary shapes. They recover their original permanent shapes from temporary deformations under external stimuli of heat, light, electricity, and solution [2]. Typically, SMPs contain at least two separate phases: a crosslink phase, which determines the permanent shape, and a switching segment, which is responsible for the temporary shape [3].

Usually, many polymers show shape memory behavior, including cross-linked PE, cross-linked ethylene-vinyl acetate copolymer, and epoxy [4]. Among them, polyurethane (PU)-based SMP offers many advantages in terms of structure designable, higher shape recoverability, a wider range of shape recovery temperature, and better biocompatibility. However, neat SMP materials suffer from relatively low thermal stability, strength, and stiffness, which hinders their applications as functional and structural materials [5,6].

It has been noticed that liquid crystalline polymers (LCPs) have unique properties in that the orientational ordering of the polymer chains in the melt phase distinguishes it from the isotropic phase [7,8]. Typically, LCPs have the virtues of a high mechanical strength at high temperatures, extreme chemical resistance, and a good weather ability, which benefits the shape memory effect [9].

It is well known that the mechanical properties of SMPs are strongly dependent on the aspect ratio of the filler and the interaction between the filler and the elastomer [10]. Coleman reports that carbon nanotubes improve the stiffness of polymethylmethacrylate [11]. Graphene oxide (GO) is a two-dimensional nanomaterial with a typical pseudo-two-dimensional structure that can provide reinforcement to the base polymer matrix [12]. Wallace *et al.* [13,14] reported highly stretchable electrodes composed of reduced GO, single-walled carbon nanotubes (SWNTs), and PU via a spray coating technique. The GO/SWCNT/PU composite electrode has potential advantages for wearable and biocompatible devices.

In this work, a novel structure of SMP is reported. Main-chain liquid crystalline PU was synthesized through *in-situ* polymerization, which improved the mechanical property of PU. Moreover, the ionic group was introduced to PU, which enhanced the compatibility of GO with the PU matrix. Furthermore, GO was added to PU to enhance the mechanical property and the shape memory performance of the PU-based SMP in terms of shape-recovery and response speed. This method presented an effective way of fabricating the GO/liquid crystalline polyurethane (LCPU) composite. The drawbacks of low thermal stability, strength, and stiffness, from which conventional SMPs suffer, can be simultaneously overcome.

## 2. Materials and Methods

### 2.1. Materials

4,4′-methylenediphenyl diisocyanate (MDI), 1,6-hexanediol (HDO), 2,2-dimethylol propionic acid (DMPA), polytetramethylene ether glycol (PTMG, *M*_n_ = 1000), *N*,*N*-dimethylformamide (DMF, 99.9 wt % purity), and graphite were obtained from Shanghai Aladdin Bio-Chem Technology Co., LTD. (Shanghai, China). PTMG was purified by dehydrated at 100 °C in a vacuum for 1 h. DMF was dried with CaH_2_ and distilled under reduced pressure prior to use. Graphene oxide (GO) was prepared from graphite powders using Hummers’ method [15].

### 2.2. Procedure of Producing GO/LCPU Composites

#### 2.2.1. Preparation of LCPU

In a three-neck round-bottomed flask equipped with a mechanical stirrer and condenser, the mixture of 5.0 mmol PTMG-1000 (5.00 g), 10.0 mmol MDI (2.50 g), and 15 mL of DMF were heated to 80 °C for 1 h in nitrogen. Then, 2.5 mmol HDO (2.95 g) and 2.5 mmol DMPA (3.35 g) were added slowly. The mixture solution was heated at 80 °C for 12 h. Films were produced by casting the product into polytetrafluoroethylene mold and allowing them to dry at 80 °C for 12 h.

#### 2.2.2. Preparation of GO/LCPU Composite Membrane

A solution casting method was used to prepare a series of GO/LCPU composite films. The procedure to prepare GO/LCPU composite films was as follows: First, a certain amount of GO was dispersed in the DMF (20 wt %) and ultrasonicated for 30 min. The amounts of GO added were 5, 10, 15, and 20 wt % of the mass of LCPU, hereafter referred to as LCPU5, LCPU10, LCPU15, and LCPU20, respectively. Then, LCPU was added, followed by stirring at 25 °C for 30 min, and then ultrasonicated for 30 min. Finally, the mixture dispersion was cast in an oven at 80 °C for 24 h forming a film layer with a thickness of 3 mm. In addition, the LCPU5, LCPU10, LCPU15, and LCPU20 are used to represent the LCPU5, LCPU10, LCPU15, and LCPU20, respectively.

### 2.3. Characterization

Fourier transform infrared (FT-IR) spectra were recorded on a Thermo Scientific Nicolet 6700-OMNI Smart Accessory Spectrum FT-IR spectrometer (Thermo Scientific, Waltham, MA, USA).

X-ray diffraction (XRD) spectra were collected on Bruker D8 Focus (BRUKER AXS, Berlin, Germany).

Differential scanning calorimetry (DSC) was performed in a range from −50 to 250 °C. The scans was performed at a heating and cooling rate of 10 °C·min^−1^ in a nitrogen atmosphere (flow = 40 mL·min^−1^).

Thermogravimetric analysis (TGA) was carried out on a TA AQ-500 (TA Instruments, Waltham, MA, USA) in the range of 25 °C–500 °C in nitrogen (flow = 50 mL·min^−1^) at a heating rate of 20 °C·min^−1^.

LC textures were observed under a polarizing optical microscope (POM) on a Leica DM4500P (LEICA, Berlin, Germany) equipped with a hot stage.

Scanning electron microscopy (SEM) was performed with field emission SEM (FESEM) S-4800 (Hitachi, Tokyo, Japan) at an acceleration voltage of 100 kV.

X-ray photoelectron spectroscopy (XPS) analyses were carried out using a Kratos AXIS-ULTRA (SHIMADZU, Kyoto, Japan).

Raman spectrum was performed with a Renishaw RM2000 confocal Raman spectrometer (RENISHAW, London, UK) with a 514-nm excitation laser.

Atomic force microscopy (AFM) was performed with Asylum Research MFP-3D Classic (OXFORD Instruments, Oxford, UK) in tapping mode.

Transmission electron microscopy (TEM) was performed with Tecnai G2 20 S-TWIN (FEI, Hillsboro, OR, USA).

Tensile tests were carried out at a room temperature (25 °C) using a Instron 5567 (INSTRON, Boston, MA, USA). A cross-head speed was set at 50 mm/min to ensure the stress–strain curves were gained to illustrate the toughness or the stored energy of the samples.

Characterization of the shape memory performance was carried out using a digital camera. All samples were annealed in an oven at 120 °C for 1 h before tests. The rectangle membrane was folded along its longitudinal center forming two small rectangle planes with an angle of 0° at 90°C and the deformation was kept at 0 °C for 10 min. Upon stopping cooling, a small angle (*A*_u_) of the sample was recovered instantaneously. Then, all samples were put into the dryer at 90 °C, and the shape recovery was recorded by camera. The shape fixity (*R*_f_) and shape recovery ratio (*R*_r_) were calculated according to Equations (1) and (2), where *A*_m_ was the angle after reheated. The angle was read from the protractor below the samples.

(1) $$R_{f}\left(\%\right)=\frac{(180^{{}^{\circ}}-A_{u})\times 100}{180^{{}^{\circ}}},\;\text{and}$$

(2) $$R_{r}\left(\%\right)=\frac{A_{m}\times 100}{180^{{}^{\circ}}}.$$

## 3. Results and Discussion

### 3.1. Characterization of GO

GO was obtained from graphite power through oxidation. The creating polar groups (carboxyl group, hydroxyl) on the surface of graphite benefitted the dispersion of GO in solution.

**Figure 1:**
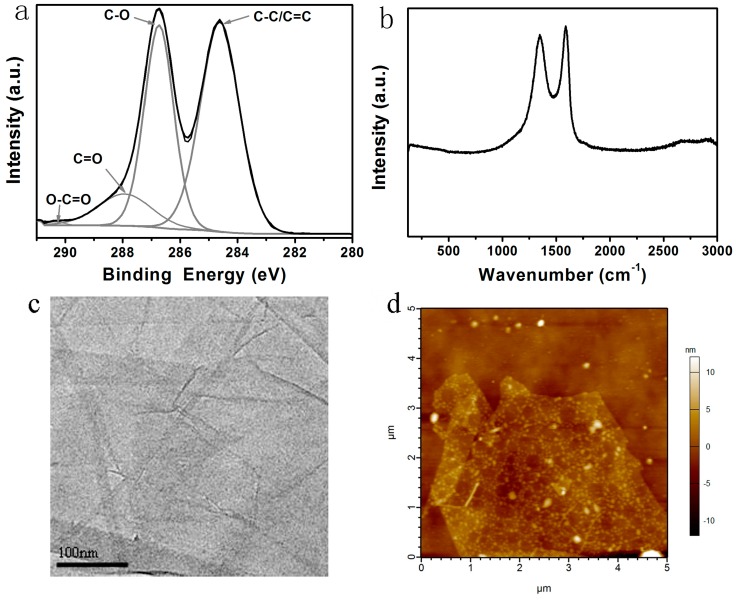
Characterization of graphene oxide (GO). (**a**) X-ray photoelectron spectroscopy (XPS) of GO film; (**b**) Raman spectrum of GO film; (**c**) transmission electron microscopy (TEM) of GO; (**d**) atomic force microscopy (AFM) of GO.

The chemical characteristics of GO were studied using X-ray photoelectron spectroscopy, as shown in Figure 1a. The C1s spectrum of the GO film showed that it consisted of two main components arising from C to O (hydroxyl and epoxy, ~286.5 eV) and C=C/C–C (~284.6 eV) groups and two minor components from C=O (carbonyl, ~288.3 eV) and O–C=O (carboxyl, ~290.3 eV) groups. The degree of oxidation of GO was measured by the surface O/C atomic ratio. From Figure 1a, the O/C atomic ratio of GO is 0.47, which is higher than the reported value of 0.44 [16].

The Raman spectrum of GO film (Figure 1b) showed two intensity peaks: the D-band (ID) around 1350 cm^−1^ and the G-band (IG) around 1580 cm^−1^. The D-band was caused by the defects and disorders in the hexagonal graphitic layers, while the G-band corresponded to an E2g mode of graphite [17]. The ID/IG ratio of GO was 0.93, which was higher than that of 0.91 reported by Zoraida [16]. It was consistent with the result of XPS. The TEM image of GO (Figure 1c) showed a flat structure. The AFM image of GO (Figure 1d) showed that the thickness of GO was about 3 nm.

### 3.2. FT-IR

**Figure 2:**
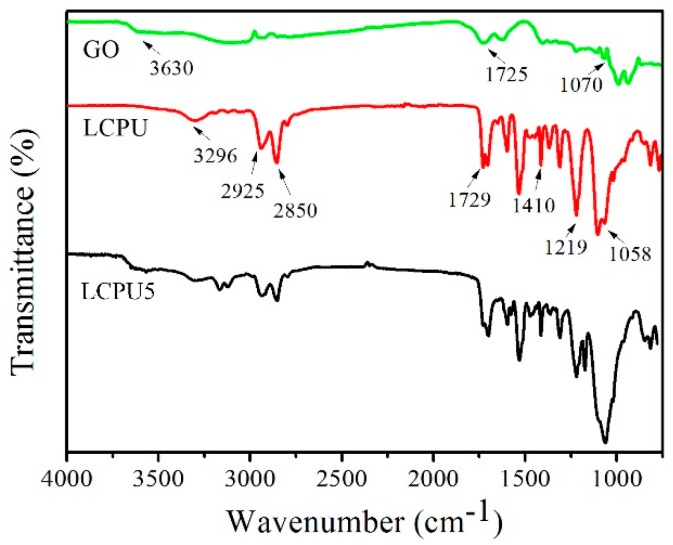
Fourier transform infrared (FT-IR) spectra of GO, liquid crystalline polyurethane (LCPU), and LCPU with 5 wt % GO (LCPU5).

The FT-IR spectra of GO, LCPU, and GO/LCPU composite films are shown in Figure 2. The spectrum of LCPU showed some typical functional groups. The most relevant bands corresponded to N–H stretching at 3174–3160 cm^−1^, which indicated that polyurethanes were capable of forming hydrogen bonds in which the N–H group of the urethane linkage was the proton donor [18]. The peaks of 2925, 2850, and 1410 cm^−1^ were assigned to the CH_2_ groups. The absence of NCO groups at 2250–2279 cm^−1^ indicated that the reaction proceeded until complete conversion of the isocyanate [19]. In addition, the spectrum of LCPU showed the characteristic C–O and N–CO–O stretching (1219–1218 cm^−1^; 1058–1056 cm^−1^) bands in polyurethanes. The peak at 1729 cm^−1^ was related to the carboxyl groups. These spectral features showed that LCPU was successfully synthesized. There are some typical features of GO shown in Figure 1c: O–H stretching at 3630 cm^−1^, C=O stretching at 1725 cm^−1^, and C–O–C vibration of epoxy at 1070 cm^−1^ [20].

### 3.3. XRD

**Figure 3:**
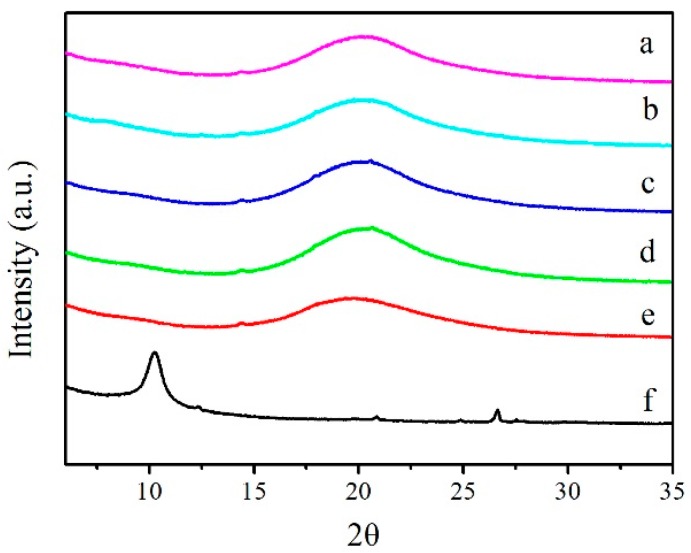
X-ray diffraction (XRD) patterns of LCPU (**a**); LCPU5 (**b**); LCPU with 10 wt % GO (LCPU10) (**c**); LCPU with 15 wt % GO (LCPU15) (**d**); LCPU with 20 wt % GO (LCPU20) (**e**); and GO (**f**).

The XRD patterns of LCPU, GO, and GO/LCPU composite films with different doping levels are shown in Figure 3. In Figure 3a, the broad peak of 2θ around 19.6° appearing in LCPU was the characteristic peak of semi-crystallization of LCPU [21].

The GO showed a strong diffraction peaks at 2θ of 12.1° (Figure 3f), corresponding to a d-spacing of 0.73 nm. It was noticed that a weak diffraction peak at 2θ of 26.8° from graphite indicated that the GO was not fully oxidized.

For the GO/LCPU composites, the peak intensity marginally increased to 2θ = 20.9° (LCPU5), 20.6° (LCPU10), 20.3° (LCPU15), and 20.3° (LCPU20), respectively. This could be related to the crystallinity caused by the GO in the LCPU matrix due to the strong nucleating effect GO has within the polymer matrix. Besides, the XRD pattern of the GO/LCPU composites showed no peak at 2θ = 12°, originating from the loss of regularity or exfoliation of the GO in the LCPU, which indicated a good dispersion of fillers in the LCPU matrix.

### 3.4. TGA

**Figure 4:**
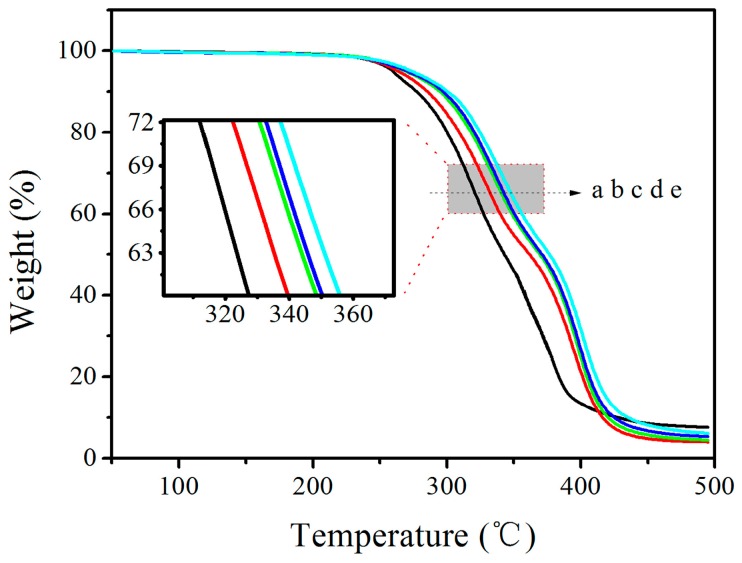
Thermogravimetric analysis (TGA) curves: LCPU (**a**); LCPU5 (**b**); LCPU10 (**c**); LCPU15 (**d**); LCPU20 (**e**).

The thermal stability of composites was characterized by TGA (Figure 4). A magnified picture of a local part was inserted for better distinctness. It clearly showed that the curves shifted towards higher temperatures after adding GO into the LCPU matrix. In order to estimate the thermal stability, the temperatures corresponding to 2% (*T*_2%_) and 50% (*T*_50%_) weight-loss of the composites were taken as the criteria. The *T*_2%_ and *T*_50%_ of LCPU20 were 243 and 377 °C increased by 5 and 34 °C, respectively, compared with that of LCPU (238 and 343 °C). Obviously, the addition of GO into the LCPU was able to significantly improve the thermal stability of the composites. This is probably owing to the “tortuous path” effect of graphene sheets, which delayed the escape of volatile degradation products and char formation as well [22].

### 3.5. DSC

**Figure 5:**
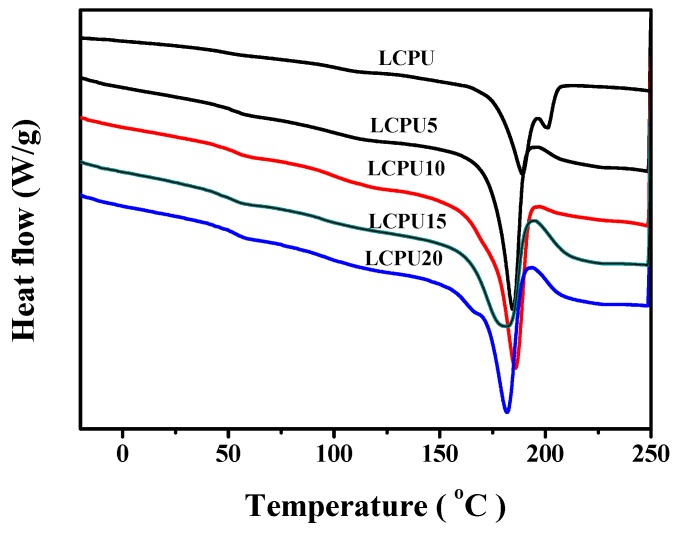
Differential scanning calorimetry (DSC) curves: LCPU, LCPU5, LCPU10, LCPU15, and LCPU20.

The dependence of the thermal transition temperatures on the LCPU and GO/LCPU composites were studied via DSC experiments. Figure 5 showed the heating DSC curves of the samples at a rate of 10 °C/min in a nitrogen atmosphere after eliminating the thermal history. Based on the heating process, all phase transition temperatures of the films are shown in Table 1. It can be seen that there were three thermal events in the range from −50 to 250 °C.

First, the single glass transition identified from Figure 5 illustrated that the monomers were placed randomly along the main chain without phase separation [23]. All phase transition temperatures of the films are listed in Table 1. The glass transition temperature (*T*_g_) of LCPU was observed at about 40.8 °C. The *T*_g_ values of the GO/LCPU composites were slightly higher than those of the LCPU. Among all samples, LCPU 20 exhibited the highest *T*_g_ of 42.8 °C. The increasing *T*_g_ of the GO/LCPU composites are attributed to the well-dispersed GO that interacted with the LCPU and hindered the relaxation of the polymer chain.

Second, the melting transition of the hard domain microcrystals was observed. Neat LCPU showed the *T*_m_ of 189.3 °C, which was higher than that of the GO/LCPU composites. In addition, the *T*_m_ of composites decreased as the GO content increased. Among them, LCPU20 showed the lowest *T*_m_ of 181.8 °C. It was supposed that the interaction between GO and LCPU improved the thermal behavior of the polymer chain due to the good thermal conductivity of GO [24]. Third, liquid crystalline mesophase-isotropic transition (*T*_i_) of neat LCPU was observed at about 200.6 °C. Meanwhile, the GO/LCPU composites did not show *T*_i_ under test conditions because of the effect of GO on the crystallinity of the LCPU matrix.

### 3.6. POM

**Figure 6:**
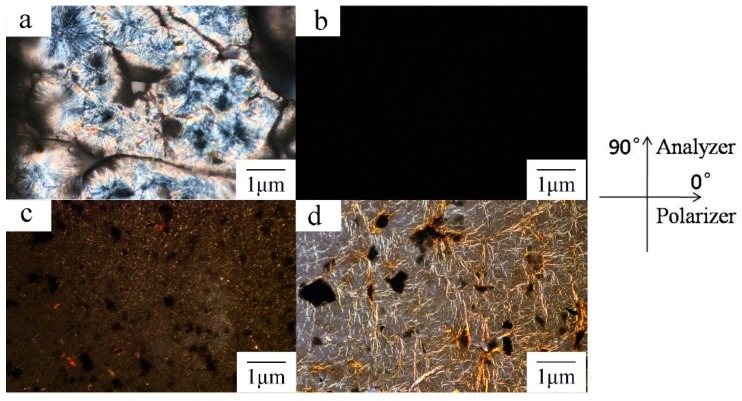
Representative polarizing optical microscope (POM) images of the texture of LCPU at 115 °C (**a**); LCPU5 at 145 °C (**b**); LCPU20 at 250 °C (**c**); and GO at 200 °C (**d**) (Magnification: 200×).

The POM observation of LCPU showed a single enantiotropic liquid crystalline phase from room temperature up to their clearing points (Figure 6a). The birefringence disappeared when LCPU was heated to 201 °C. Moreover, the texture appeared again when the sample was cooled down to 230 °C. However, the POM images of the LCPU5 composite film did not show any texture when the sample was heated from 25 to 250 °C. Further, the image turned to black completely under POM at 201 °C without any birefringence (Figure 6b). A similar texture with that of LCPU5 appeared for samples LCPU10 and LCPU15 (images not shown). It is interesting that the LCPU20 showed a texture that was different with that of LCPU. Moreover, birefringence of the GO film was observed, which was quite different from LCPU. We deduced that different birefringences influenced each other, and the composition of the composite was a key factor. The interaction mechanism of the component and the birefringence in the composite needs further investigation.

### 3.7. SEM

**Figure 7:**
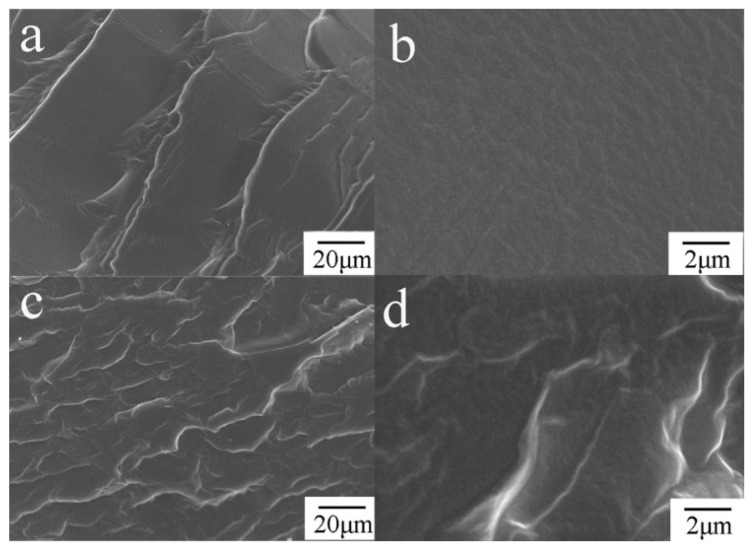
Field emission scanning electron microscopy (FESEM) images of LCPU (**a**,**b**) and LCPU5 (**c**,**d**).

Figure 7 showed the SEM morphology obtained from the fracture surface of the LCPU and LCPU5. The neat LCPU showed a smooth fracture surface, while the GO/LCPU composite film appeared to have a rough surface due to the interaction between GO and the LCPU matrix, which would benefit the stiffness of the GO/LCPU composite. Quaresmin and Nadiv [25,26] suggested that the enforcement of the mechanical property in the nanofiller-polymer composite was attributed to the toughening mechanism of graphene by a crack bifurcation-deflection mechanism. The carbon nanotube composites had a rough fracture surface because that carbon nanotube was departing from the polymer matrix. We deduced that, for our GO/LCPU composite, the interaction between GO and LCPU was responsible for the rough fracture surface. Moreover, the interaction above enhanced the mechanical property of the GO/LCPU composite films.

### 3.8. Mechanical Properties

**Figure 8:**
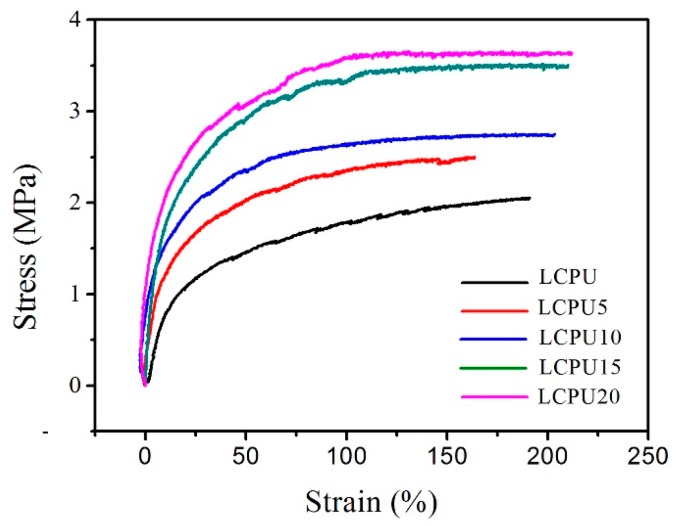
Stress–strain curves of the pristine LCPU and its composites at room temperature. Stretching rate was set at 50 mm/min.

**Figure 9:**
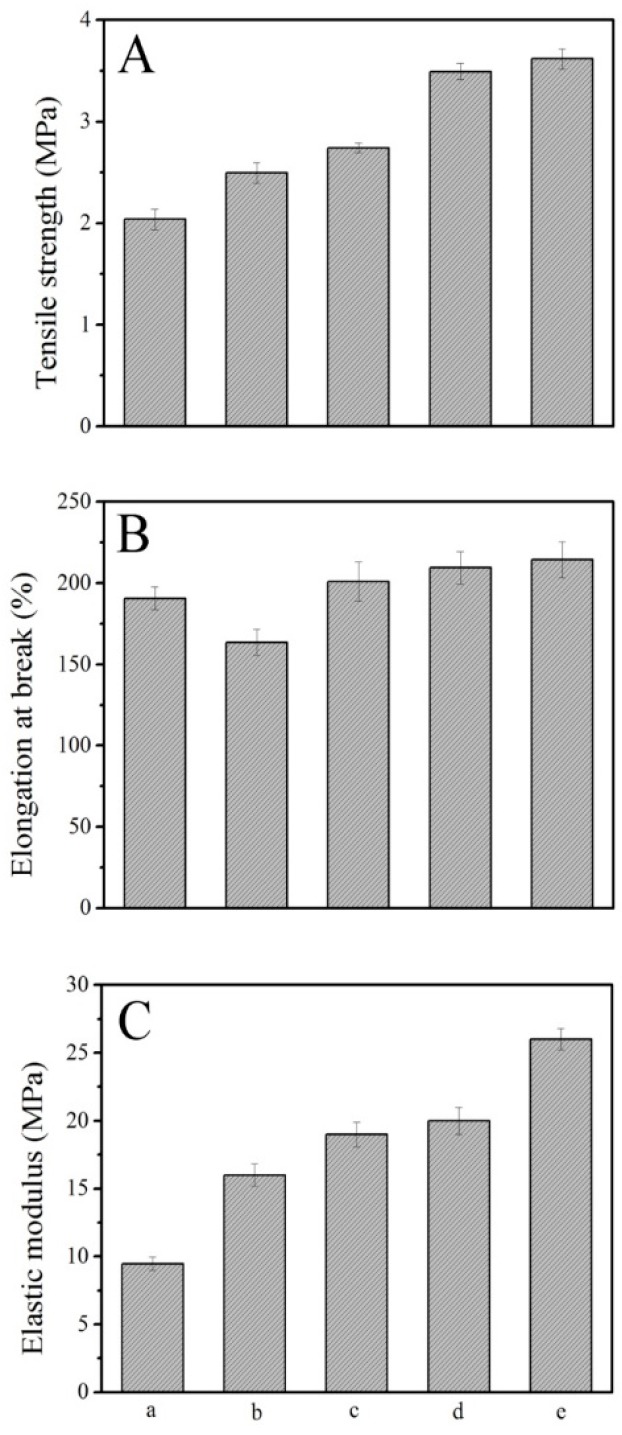
Tensile strength (**A**); elongation at break (**B**); and elastic modulus (**C**); LCPU (**a**); LCPU5 (**b**); LCPU10 (**c**); LCPU15 (**d**); LCPU20 (**e**).

In the GO-reinforced polyurethane composites, their mechanical properties were significantly influenced by the GO dispersion status and interfacial interaction between GO and the matrix [27]. The mechanical properties of the LCPU and GO/LCPU composite films were evaluated using tensile testing as a function of the GO content. The representative stress–strain curves of LCPU and GO/LCPU composite films are shown in Figure 8. The mechanical property in terms of tensile strength, modulus, and elongation at break of the LCPU-based film were markedly enhanced by the incorporation of GO (Figure 9). The most effective enhancement was obtained for the LCPU20 composite film. Its tensile strength, elongation at break, and elastic modulus were 3.618 MPa, 214.32%, and 26 MPa, corresponding to increases by 77.5%, 12.4%, and 174.8% compared with those of neat LCPU. The remarkable improvements of the mechanical properties in the GO/LCPU composites were due to the good dispersion of GO in the LCPU matrix and the interfacial interactions between GO and LCPU. Some works have demonstrated that the mechanical property of PU improved due to the good dispersion and bonding of graphene sheets with the PU matrix [28,29]. In our case, the strong interaction of PU and GO is attributed to the carboxyl group in the main chain of LCPU that was able to form hydrogen bonding with GO, which leads to the effective load deliver from the LCPU to GO under external stress [30]. Furthermore, the enhancement of the tensile strength and elongation at break was able to enlarge the area under the curve, which was crucial in shape memory because the area was the strain energy that is stored when stretching, and it stimulated strain recovery upon the release of stress in its rubbery state [31]. As shown in Figure 8, the area under the curve of LCPU20 was much higher than that of pristine LCPU, which means that LCPU20 had a much greater force for shape recovery in its rubbery state.

### 3.9. Shape Memory Property

**Figure 10:**
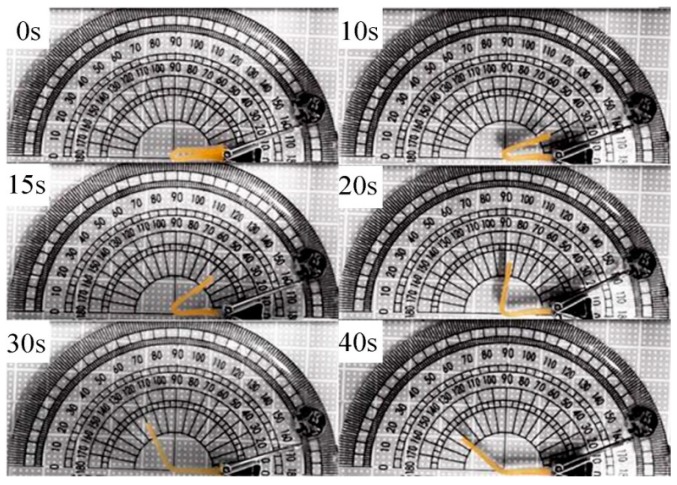
Recovery photos at different times of LCPU at first circle.

The shape memory effect of the LCPU and GO/LCPU composites was investigated. Rectangular specimens were deformed via folding at 90 °C for a few minutes, followed by rapid quenching in cold water. No apparent recovery was seen after the deformed sheet was kept in air for 2 h. Then, the sample was put into 90 °C water and the deformed SMP sheet started to return to its original shape. Figure 10 showed the photos of the shape memory effect of the LCPU film. It could be seen that the folded SMP started to recover its original shape at 90 °C. A series of GO/LCPU composite films were tested for their shape memory effect. The shape fixity and shape recovery ratio for four cycles of the LCPU and GO/LPU composite films were listed in Table 2. Table 3 showed the shape recovery rate of all the samples.

As shown in Table 2, the shape fixity ratio *R*_f_ of composite samples decreased as the GO content increased; the value dropped from 98% of LCPU to 92% of LCPU20 in the first cycle. Nevertheless, all samples maintained their *R*_f_ values in the following three cycles. The shape recovery ratios (*R*_r_) of the composite samples were similar. The *R*_r_ values of neat LCPU and LCPU20 were similar in the first cycle. All the *R*_r_ values slightly decreased as cycle number increased. Shanmugharaj *et al.* [32] reported that the recovery ratio dropped in cycles for carbon nanotube–PU nanocomposites because of the formation of frozen-in crystals due to an increase in the orientation of molecular segments in the direction of external force on repeated cycling.

Furthermore, the shape recovery rate of samples markedly increased with the GO loading increase, as shown in Table 3. The shape memory behavior was the result of a phase transition of polymer, which was caused by heat change due to the thermal conductivity. GO had a high thermal conductivity, which is attributed to the high-speed shape recovery response of the GO/LCPU composite film [33].

## 4. Conclusions

A novel structure of shape memory polymer (SMP) with main-chain liquid crystalline polyurethane (LCPU) that contains a large polar group was synthesized. A graphene oxide (GO)/LCPU composite film was fabricated using the solution casting method. The mechanical properties of the composite in terms of tensile strength, Young’s modulus, and the elongation improved due to the interaction between the GO and the PU matrix. Furthermore, the good thermoconductivity of GO contributed to the fast shape memory response speed of the GO/LCPU composite.

## Figures and Tables

**Table 1:** Thermotropic properties of LCPU and its composites.

Sample	*T*_g_ (°C)	*T*_m_ (°C)	*T*_i_ (°C)
LCPU	40.8 ± 0.6	189.3 ± 0.2	200.6 ± 0.1
LCPU5	41.9 ± 0.7	188.0 ± 0.2	-
LCPU10	42.7 ± 0.5	185.7 ± 0.3	-
LCPU15	42.1 ± 0.6	181.9 ± 0.2	-
LCPU20	42.8 ± 0.5	181.8 ± 0.3	-

**Table 2:** Shape fixity and shape recovery ratio of LCPU and GO/LCPU nanocomposites.

Cycles	LCPU		LCPU5		LCPU10		LCPU15		LCPU20	
	*R*_f_	*R*_r_	*R*_f_	*R*_r_	*R*_f_	*R*_r_	*R*_f_	*R*_r_	*R*_f_	*R*_r_
1	98	99	96	100	95	99	92	99	92	100
2	98	99	95	99	95	98	93	98	93	99
3	97	98	95	96	95	97	93	98	93	99
4	98	97	96	96	94	97	93	98	93	98

**Table 3:** Average shape recovery rate of all the samples.

Cycles	LCPU (°/s)	LCPU5 (°/s)	LCPU10 (°/s)	LCPU15 (°/s)	LCPU20 (°/s)
1	2.6	5.6	6.3	8.6	9.2
2	2.6	4.5	5.9	8.1	8.9
3	2.4	4.9	5.5	6.8	7.8
4	2.3	4.6	5.3	6.0	7.1
